# Exploring the Diagnostic and Therapeutic Pathways of Women with Dyspareunia: A Mixed-Methods Study

**DOI:** 10.3390/jcm15020787

**Published:** 2026-01-19

**Authors:** Joanna Wojtas, Zofia Sotomska, Marek Murawski, Magdalena Emilia Grzybowska

**Affiliations:** 1 Department of Gynecology, Obstetrics and Neonatology, Medical University of Gdańsk, Smoluchowskiego 17, 80-214 Gdańsk, Poland; joanna.wojtas@gumed.edu.pl; 2 Independent Team of Physiotherapists, University Clinical Center, al. Zwycięstwa 30, 80-219 Gdańsk, Poland; zofia.sotomska@gumed.edu.pl; 3 Department of Clinical Physiotherapy, Medical University of Gdańsk, Zwycięstwa 30, 80-219 Gdańsk, Poland; 4 Department of Gynecological Surgery and Oncology, Wroclaw Medical University, 50-367 Wroclaw, Poland; marek.murawski@umw.edu.pl; 5 Department of Obstetrics and Gynecology, Gynecologic Oncology and Gynecologic Endocrinology, University Clinical Center, Dębinki 7, 80-952 Gdańsk, Poland

**Keywords:** dyspareunia, knowledge, pain, sexual dysfunction, treatment, mixed-methods study, women’s health

## Abstract

**Background/Objectives**: This study explores the diagnostic and management pathways for dyspareunia in women seeking specialist care, focusing on gynecologists’ feedback and women’s perceptions of their experience. **Methods**: An online survey was conducted among 225 sexually active women to explore their perceptions of dyspareunia, its impact on relationships, and experiences with healthcare feedback, diagnosis, and treatment. The Numeric Rating Scale (NRS) for pain assessment and the Female Sexual Function Index (FSFI) were used. Gynecologists’ feedback was classified as positive, neutral, or negative based on its influence on the therapeutic pathway. **Results**: Of 78 women reporting dyspareunia, 12 with pain level ≥5 on NRS were selected for in-depth analysis. The mean pain score was 7.0 ± 1.53, with symptoms lasting from several months to over two years and occurring during most sexual encounters. The mean FSFI score was 24.86 ± 4.54, with half of the participants scoring within the sexual dysfunction range. Qualitative findings revealed frequent dismissive responses from healthcare professionals and limited access to appropriate management. Common self-management strategies included changing sexual positions and using lubricants, while half of the participants had not undergone a formal diagnostic process. Most frequent diagnoses were hormonal disorders and recurrent genital tract infections, and women were advised to undergo pharmacological treatment. Half of the participants were unaware of the possibility of physiotherapeutic management. **Conclusions**: Women with dyspareunia often face an inadequate diagnostic and therapeutic process. The care received is often insufficient and not aligned with a biopsychosocial model.

## 1. Introduction

Dyspareunia is one of the most common complaints among female sexual dysfunctions. It is a recurrent, persistent pain or discomfort associated with attempting or having sexual intercourse itself, and it is not always linked to any tissue pathology [1,2]. Although dyspareunia occurs in up to 21% of women, it is under-reported, with only half of women reporting symptoms to healthcare professionals [3,4]. It is a complex condition involving biological, psychological, and social factors [5,6]. Psychogenic causes of dyspareunia include childhood sexual abuse, feelings of guilt or shame about sexual intercourse, and difficulty achieving sexual arousal. Somatic causes include pelvic inflammatory disease, vulvodynia, vestibulodynia, genitourinary syndrome of menopause (GSM), hypoestrogenism associated with lactation, and endometriosis [7]. Increased pelvic floor muscle tone, although not always associated with pain, has been suggested to be an important and common component of female sexual dysfunction [8].

While the classification of dyspareunia into deep (pain localized within the pelvis) and superficial (pain experienced in the vulvar region) subtypes is widely used, it is argued that such distinctions may fragment the understanding and management of pain as a multidimensional, holistic experience [9]. In the Fifth Edition of the Diagnostic and Statistical Manual of Mental Disorders (DSM-5), the previous diagnostic categories of dyspareunia and vaginismus (characterized by involuntary vaginal muscle contraction) were replaced by a broader diagnosis: Genito-Pelvic Pain/Penetration Disorder. This transition reflects a more integrated approach to the clinical presentation [10]. Due to the diverse etiologies involved, treatment strategies are tailored to the underlying causes and specific examination findings. Effective treatment requires medical knowledge combined with an understanding of the psychosexual factors affecting each patient [11]. Interventions for dyspareunia include sexual education, psychotherapy, topical and systemic pharmacotherapy, physical therapy, and surgery (e.g., hymen incision) [1,12,13]. Only half of women with sexual dysfunction seek professional help [3,4]. Help-seeking behavior varies by age, culture, and type of sexual problem. The psychological distress associated with sexual dysfunction has been identified as primary motivator for seeking medical assistance [14]. Conversely, significant barriers—including shame, the normalization of symptoms, and lack of confidence in medical efficacy—remain substantial obstacles to care [15].

Although available data remain limited, evidence has been documented describing the challenges associated with dyspareunia and other sexual dysfunctions among young women and those with endometriosis, as well as the barriers encountered during help-seeking behaviors [16,17,18]. It is suggested that a deficit in public health information regarding dyspareunia, coupled with a clinical reluctance to address sexual health, may lead many to significant treatment delays, potentially resulting in adverse biopsychosocial consequences [16]. Furthermore, the importance of directly involving informal support networks, comprising partners, family, and friends, in healthcare management is emphasized. Given the prevalence of social media, the development of professionalized online support networks is proposed to mitigate the risks of misinformation or inadequate support [18].

This study examines the clinical pathway of diagnosing and managing dyspareunia in women seeking specialist care. It assesses the nature of feedback provided by healthcare professionals and analyzes the progression from initial symptom presentation to the implementation of therapeutic interventions. Additionally, this study explores women’s perceptions and attitudes toward their experience of dyspareunia.

## 2. Materials and Methods

### 2.1. Study Design

A mixed-methods study was conducted to explore the treatment experiences of women with dyspareunia. Data were collected through an online Google survey distributed on a social media platform (Facebook) from January to April 2020. The study employed a convergent design [19]. The quantitative component described the study population, identified patterns and frequencies, and served as a reference for the existing literature. The qualitative component addressed “how and why?” questions. It examined the participants’ experiences and perceptions, aiming to understand the meanings attributed to events, the contextual factors that influence them, and the processes that lead to specific outcomes. Quantitative and qualitative data were collected simultaneously, analyzed separately, and integrated during the interpretation through side-by-side comparison. This integration enabled the examination of numerical trends alongside participants’ narratives, providing a comprehensive understanding of dyspareunia and related health care experiences. The study was approved by the Independent Bioethics Committee for Scientific Research at the Medical University of Gdańsk, no. NKBBN/537/2019-2020. The Declaration of Helsinki was followed. Respondents were informed about the confidentiality of the collected data and the purpose of its use.

### 2.2. Sample Participants

**Figure 1:**
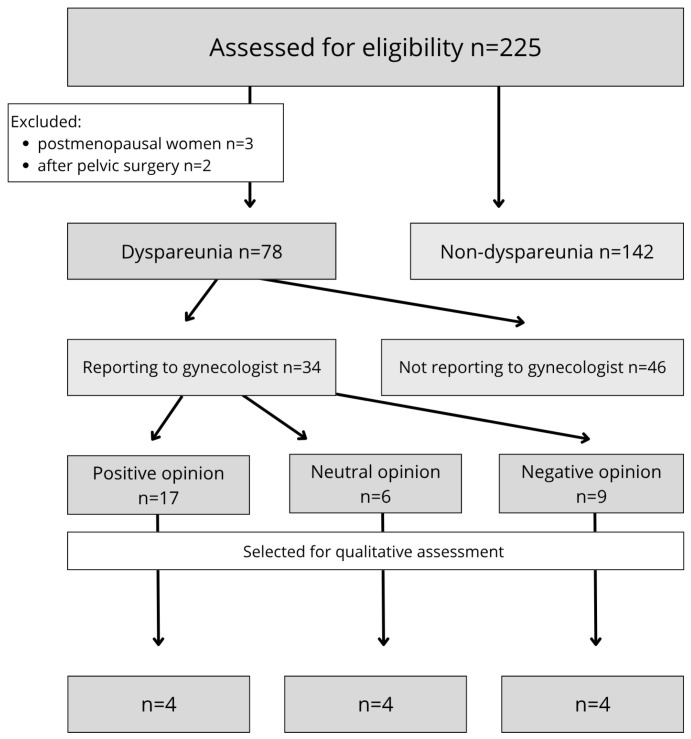
Flowchart of data selection in the study.

A total of 225 non-pregnant, sexually active adult women (sexual intercourse at least once a year) completed the anonymous survey. For qualitative analysis, data from 12 participants who reported significant pain during intercourse, defined as a score ≥ 5 on the Numerical Rating Scale (NRS), were selected. For participants meeting the inclusion criteria, it was verified whether the open-ended question regarding the gynecologist’s opinion about their symptoms had been answered. If a medical opinion was referenced, it was evaluated for diagnostic and therapeutic relevance. Responses acknowledging the problem and proposing treatment were classified as positive, whereas responses that were dismissive, inconsistent with current clinical recommendations, or offensive were categorized as negative. Responses stating a fact without suggesting any form of treatment were deemed neutral. Each case was discussed by three authors (JW, ZS, and MEG) to reach a consensus. Figure 1 shows the data selection process.

### 2.3. Questionnaire

The survey included questions about sociodemographic and medical data, information about sexual intercourse, and dyspareunia symptoms. The questionnaire was developed based on the literature addressing population-level sexual frequency, dyspareunia help-seeking behavior [3,4], and the etiology of dyspareunia-related dysfunctions [7,8]. Respondents were asked to describe the feedback received from the professional to whom they reported their symptoms. Participants reported their perceptions of dyspareunia: whether it was an embarrassing issue or a dysfunction, and whether it negatively affected their relationships. Table 1 shows the detailed questions from the survey along with the possible responses. The validated Polish version of the Female sexual Function Index (FSFI) was used to assess sexual function [20]. The FSFI includes 6 domains: desire, arousal, lubrication, orgasm, sexual satisfaction, and pain. A score of ≤26.55 was considered the cutoff point for sexual dysfunction.

### 2.4. Analysis

Content analysis was utilized as the overarching approach used for data analysis. Mean, standard deviation, and frequency for quantitative and qualitative variables were calculated for basic group characteristics. For the qualitative analysis, two authors (JW and ZS) independently reviewed the data, identified recurring themes, and coded the collected information to appropriately categorize it. In the event of difficulty or disagreement, a third author (MEG) was consulted to provide additional input and reach consensus. The qualitative analysis was not designed to examine differences or similarities between participants. Instead, individual cases were described below with a focus on help-seeking behavior and treatment. The results were presented as twelve case descriptions based on the following framework: age, NRS and FSFI scores, self-assessment of the condition, incidence, impact on relationship, gynecologist’s opinion, and previous treatment history.

## 3. Results

A total number of 225 women participated in the primary study and fully completed the questionnaire. The mean age and BMI of participants were 27 ± 7.1 years and 22.4 ± 3.4 kg/m^2^, respectively. Among those reporting dyspareunia (*n* = 78; 34.7% of the sample), the average pain intensity, measured using the NRS, was 5.5 ± 2.12. The mean total FSFI score was 22.7 ± 11.7 and 21.7 ± 8.3 in women without and with dyspareunia, respectively.

Out of participants diagnosed with dyspareunia who consulted a gynecologist, a purposive sample of 12 women was selected for the qualitative analysis. Their mean age was 26 ± 3.51 years, mean BMI was 21.82 ± 3.58 kg/m^2^, and mean NRS pain intensity was 7 ± 1.53. The mean FSFI score was 24.86 ± 4.54, with 58.3% of participants scoring ≤26.55 (Table 2).

Findings indicated that dyspareunia represented a considerable concern within this cohort, with more than half of the women experiencing significant pain during every sexual encounter. Table 3 summarizes the perspectives of the consulting physicians, Table S1 (Supplementary Materials) provides a detailed overview of the qualitative study participants.

### 3.1. Respondent No. 1—Negative Feedback

Woman aged 25 years with an FSFI score of 24; the highest scored domain: pain 5.6; the lowest scored domain: lubrication 2.1; NRS score 5. Reported that dyspareunia is a shameful topic and a dysfunction. She had sexual intercourse once a week and experienced pain every time. The pain had been present from the beginning of her sexual activity, and was described as burning, sharp, and sudden. It negatively affected her relationship with her partner. The gynecologist’s opinion was: “Intercourse is possible, so from a medical point of view, there is no problem”. She was diagnosed with vaginismus and an insusceptible hymen. She underwent hymen incision surgery with no improvement. Treatments that she underwent for dyspareunia were psychological counseling, surgery, local lubrication, and sexual position change.

### 3.2. Respondent No. 2—Negative Feedback

Participant was 31 years of age with an FSFI score of 25.3; the highest scored domain: sexual satisfaction and arousal—5.6; the lowest scored domain: pain 2.8; NRS score 7. She described painful intercourse as an embarrassing problem and a disorder. Reported having pain during every instance of sexual intercourse since she began having intercourse. Described the symptom as “chronic, stabbing” and had intercourse once a week. The symptoms negatively affected her relationship with her partner. She described her visits to specialists as follows: “I met with the same dismissive opinions. That it wasn’t a gynecological problem or that it was apparently just my beauty. Of course, there was also the suggestion to change my partner for a better one (implicitly in bed)”. Ultimately, she was not diagnosed, and the measures she took included a change in sexual position, local lubrication, and a visit to a pelvic floor physiotherapist.

### 3.3. Respondent No. 3—Negative Feedback

Participant aged 22 years; FSFI score was 27.8; highest scored domain: sexual satisfaction 5.6; lowest scored domain: pain 2.8; NRS score 7. Evaluated painful intercourse as a dysfunction and as an embarrassing problem. Experienced painful intercourse several times a week, with acute, sudden, and stabbing characteristics. The pain appeared sometime after she initiated intercourse, with no identifiable cause. However, the experience did not affect her relationship with her partner. She consulted a gynecologist about her complaints, after which she tried topical lubrication and changing sexual positions.

### 3.4. Respondent No. 4—Negative Feedback

Woman aged 26; FSFI score 24.3; the highest scored domain: sexual satisfaction 5.6; the lowest scored domain: pain 1.2; NRS score 10. Did not consider dyspareunia as a pathology or an embarrassing topic. She had experienced pain with intercourse since she started having intercourse and experienced pain every time. She described the pain as follows: “It’s as if someone is forcefully trying to get through something they can’t fit through. My husband also says he feels the vaginal muscles squeezing involuntarily, feeling as if he has hit a wall.” Dyspareunia has not hurt her relationship. She was not diagnosed with any condition. She had several visits to a physiotherapist, although she did not obtain an opinion on the subject, and tried changing sexual positions and also the use of topical lubrication and topical analgesics.

### 3.5. Respondent No. 5—Positive Feedback

Woman aged 28; FSFI score was 18.6; the highest scored domain: sexual satisfaction 4.6; the lowest scored domain: pain 1.6; NRS—10. Considered dyspareunia both a disorder and an embarrassing issue and described the pain as chronic. She had experienced pain with every instance of sexual intercourse since she started having intercourse and had intercourse once a week; however, in her opinion, dyspareunia did not affect her relationship. She was diagnosed with vulvodynia and recommended electrotherapy, laser therapy, and a visit to a pelvic floor physiotherapist. She followed the recommendation to see the physiotherapist but also took medication, used topical lubricants, tried changing sexual positions, and benefited from a visit to a psychologist.

### 3.6. Respondent No. 6—Positive Feedback

Woman aged 25 years old; assessed symptoms using FSFI with a score of 26.8; the highest scored domain: orgasm 5.6; the lowest: pain 2.8; NRS 8. Considered pain during intercourse a non-physiological sensation and said it was embarrassing. The pain had been felt with every instance of intercourse since she started having intercourse. Described the pain as “sudden, stabbing, sharp”. She had intercourse once a month, and dyspareunia had reduced the quality of her relationship. The gynecologist found no anatomical abnormalities and recommended consultation with a pelvic floor physiotherapist. Ultimately, she used only topical lubricants to reduce the pain and changed her sexual positions.

### 3.7. Respondent No. 7—Positive Feedback

Woman, aged 24 years; FSFI score 31.3; the highest domain: sexual satisfaction 6; the lowest domain: pain 4.4; NRS score 7. Claimed that dyspareunia is abnormal but not embarrassing. Did not specify when the painful intercourse started, and had been experiencing it for about 2 years, sporadically. She described the pain as “dull, unspecific.” The symptom has not negatively affected her relationship. The specialist she visited stated that treatment was necessary and made a diagnosis of chronic reproductive tract infections. Her treatment included pharmacological agents, changing sexual positions, and topical vaginal lubrication.

### 3.8. Respondent No. 8—Positive Feedback

A woman aged 24; FSFI score was 18.6; the highest scored domain: sexual satisfaction 4.4; the lowest scored domain: desire 1.8; NRS 5. Described pain with intercourse as unphysiological and embarrassing. She had been experiencing pain for several years, since the initiation of intercourse and during every preceding intercourse. Described the complaints as “sharp and sudden.” Dyspareunia did not negatively affect her relationship. A specialist diagnosed the increased tone of the pelvic floor and gluteal muscles. She undertook topical lubrication, low back area massages, and manual therapy of the pelvic floor.

### 3.9. Respondent No. 9—Neutral Feedback

Woman 24 years old; FSFI score was 28.3; the highest domain score (sexual satisfaction) was 5.6, the lowest domain score (pain) was 3.6, and NRS score was 7. Described painful intercourse as a disorder and as an embarrassing topic. Experienced pain every 2–3 instances of intercourse and engaged in intercourse several times a week. The pain occurred at the beginning of intercourse and was termed “burning pain”. Dyspareunia has negatively affected her relationship. The only answer she received from the specialist was that he saw nothing to worry about. She chose topical lubricants as her pain-management method.

### 3.10. Respondent No. 10—Neutral Feedback

Woman aged 35; FSFI was 16.6; the highest domain scored: lubrication 5.1; the lowest-scoring domain: orgasm 1.6; NRS 9. Dyspareunia was an embarrassing topic and a disorder for the respondent. During intercourse, which occurred once a month, she experienced severe pain every 2–3 acts of intercourse. This began at the onset of intercourse, was described as “burning, acute, sudden, chronic, rasping, and widespread”, and interfered with the subject’s relationship. She was diagnosed by a specialist with chronic genital tract infections and urinary incontinence. She mentioned the following: “Delicate structure, shallow distribution of blood vessels, anteflexion of the uterus, cervical ectopy.” She implemented pharmacotherapy, changing sexual position, local lubrication, consulting a psychologist, surgical treatment, partner examination (not specified), and changing intercourse technique and frequency.

### 3.11. Respondent No. 11—Neutral Feedback

Woman aged 24; FSFI score was 30.5, with the highest domain (sexual satisfaction and lubrication) scoring at 6, and the lowest domain (orgasm) scoring at 3.6, with an NRS of 5. Dyspareunia for the respondent was not an embarrassing subject, but she characterized it as a disorder. Pain appeared from the very beginning of sexual activity and occurs once every 2–3 acts of intercourse. Pain was described as “sudden, sharp and stabbing pain”, which ultimately did not negatively affect the relationship. The specialist diagnosed uterine myomas and hormonal disorders. She applied drug treatment and attempted to change sexual positions.

### 3.12. Respondent No. 12—Neutral Feedback

Woman aged 24; FSFI score was 26.2; the highest domain score was 6 (sexual satisfaction) and the lowest domain score was 1.6 (pain), with an NRS of 7. Did not evaluate dyspareunia as something abnormal; instead, she treats it as an embarrassing topic. The pain appeared with the onset of intercourse, several years ago, and now appears as “a burning and stabbing sensation”, which occurs every 2–3 acts of intercourse without negatively affecting the relationship. No diagnosis was made for the respondent, and she had tried using topical vaginal lubrication to reduce the pain.

## 4. Discussion

Of the 225 women surveyed, 78 (34.7%) reported experiencing painful intercourse. Due to the recruitment method (a social media-based survey), the study sample was predominantly composed of younger women, with a mean age of 26 ± 3.51 years. No significant age difference was observed between participants with and without dyspareunia. In the literature, the prevalence of dyspareunia among women aged 20–30 years ranges from 8% to 19% [3,21], suggesting a higher prevalence within this study’s cohort.

Women with dyspareunia reported high mean pain intensity on the NRS, with half of them experiencing pain during every sexual encounter. These findings align with the existing literature, where women with dyspareunia typically report moderate to severe pain (NRS scores > 5) [22,23]. For instance, Schvartzman et al. observed a mean pain score of 7.7 ± 1.6 on the Visual Analogue Scale (VAS) among perimenopausal women, while even higher mean VAS scores of 9.03 ± 0.86 and 8.34 ± 0.97 have been reported in younger cohorts [3,22]. Regarding help-seeking behavior, less than half of the symptomatic women discussed the issue with a gynecologist. Approximately one-fifth reported it to a pelvic floor physiotherapist, and a negligible number consulted a psychologist. Ultimately, only one-third of the participants underwent treatment for their symptoms. Notably, our qualitative analysis was restricted to women who had discussed their condition with a gynecologist. Globally, the disclosure rate of sexual dysfunction varies by region, ranging from 16.7% to 25.2%, with the highest reporting rates in Central and South America (up to 40%), followed by Europe, and the lowest in East Asia (8.5%) [4]. Help-seeking behavior beyond gynecological care is relatively rare; only 1–8% of women consult a psychiatrist, psychologist, or marriage counselor, whereas 8.7% to 15.3% approach pharmacists when starting treatment [4].

The mean FSFI score was 24.9, with more than half of the participants scoring below the cutoff point, indicating sexual dysfunction. As anticipated, the lowest scores were observed in the pain domain. These findings align with previous studies. Donaldson et al. reported a mean FSFI score of 22.34 ± 3.71 among young women with sexual dysfunctions [16]. While FSFI scores typically vary with age, the presence of any sexual dysfunction consistently lowers the total score. In women aged 18–55 with sexual dysfunction, the mean FSFI score was 21.59 ± 6.58 [20]. Dąbrowska-Galas et al. reported that among middle-aged Polish women (mean age 52 ± 5.3), 69.73% scored below the FSFI cutoff, with the lowest scores observed in the sexual desire domain (2.9 ± 1.49), and the highest in sexual satisfaction (3.87 ± 1.8) [24]. In our cohort, however, despite reporting high pain levels, participants rated sexual satisfaction as their highest-scoring domain.

The diagnosis of dyspareunia typically relies on a comprehensive clinical assessment, including detailed history-taking, physical examination, and, where indicated, additional investigations such as ultrasound imaging or biopsy. In the present study, it was observed that a thorough diagnostic approach was applied inconsistently, with only a subset of participants undergoing a complete evaluation. Based on patients’ reports, utilized treatment strategies included psychological counseling, surgery, local lubrication, physiotherapy, pharmacotherapy, partner evaluation, and modifications to intercourse technique, position, and frequency. However, the therapeutic interventions used in our study group did not fully align with current clinical recommendations. While most participants received pharmacological treatment, lubricants, or made positional adjustments, the literature advocates for a multimodal approach, including physiotherapy, biopsychosocial care models, and shared decision-making [3,9,10,11,12,13,25,26]. Similarly, gaps in evidence-based care have been documented in large-scale studies. Dumas et al. analyzed data from over one million women in the U.S. (mean age 41.3 ± 14.8) diagnosed with dyspareunia. They reported that only 6.8% used vaginal estrogen and 3% were prescribed oral medications such as gabapentin, ospemifene, or venlafaxine. Notably, 18.7% received benzodiazepines or opioids, while physiotherapy was offered to just 0.07% of women [27]. Current guidelines emphasize that dyspareunia management should be multidisciplinary, rooted in the biopsychosocial model, and conducted by clinicians with expertise in chronic pelvic pain [27]. Pelvic floor physiotherapy is a widely supported first-line treatment, demonstrating effectiveness in most cases [28]. Cognitive-behavioral therapy is also beneficial, particularly for reducing anxiety and fear associated with intercourse-related pain, and is strongly recommended, especially as a part of a multimodal approach in provoked vestibulodynia [25].

In our study, gynecologists’ responses to patient complaints were categorized as positive, neutral, or negative. Consistent with findings in the literature, many of these responses were found to be inadequate and often misaligned with psychologically informed practices and the biopsychosocial model of care. This indicated a need for more robust algorithms and clinical recommendations that encompass both biological and contextual factors [29,30,31]. Women who experienced symptoms most often consulted a gynecologist. It was observed that not all participants recognized pain during intercourse as a clinical abnormality based on their own knowledge. Many also undertook independent measures to manage the situation, such as changing sexual position or using lubricants. In the study by Pithavadian et al. [18], it was observed that help-seeking behavior among women experiencing penetration difficulties was only initiated upon the realization that the symptoms were abnormal. As demonstrated by the present results, this was not always immediately evident. Moreover, in some cases, participants from the mentioned study were not properly diagnosed and faced challenging medical experiences. Consistent with our respondents’ accounts, this study identified similar themes such as missed diagnoses and the invalidation of their experiences. Examples of overlapping participants’ statements include: “I was repeatedly told it might still be endometriosis and spent a lot of time and money consulting different specialists. It was only the pelvic floor therapists who properly examined me and diagnosed pelvic floor dysfunction and vaginismus, which had previously been overlooked” or the comment one respondent achieved from a gynecologist: “Oh, it may just be you know, a lot of women have a low pain tolerance. It just might be the case for you” [18]. This illustrates that, at times, the pathway to proper diagnosis can be long and complicated.

Psychologically-informed practice emphasizes a patient-centered approach that integrates patients’ beliefs, expectations, and emotional experiences into the development of individualized treatment plans. A key component of this model is the clinical interview, which fosters open communication, trust, and mutual understanding [32,33]. Margalit et al. demonstrated that adopting a biopsychosocial framework not only enhances patient satisfaction but also has the potential to lower healthcare costs [32,33].

## 5. Conclusions

Dyspareunia may represent a deeply distressing condition that may have a considerable impact on intimate relationships. In the cases described, women frequently perceived dyspareunia as a shameful topic, yet they elected to discuss the issue with a specialist. Some participants introduced self-initiated modifications in their daily lives or sexual function in an effort to alleviate symptoms. Participants’ experiences in this explanatory qualitative study suggest that, in some cases, diagnostic and therapeutic approaches felt insufficient. Low help-seeking rates, together with negative experiences within health care setting, may constitute barriers to ongoing care and increase the risk of treatment discontinuation. Although these findings are not generalizable, they emphasize the potential value of interdisciplinary collaboration among gynecologists, sexual health therapists, psychologists, and physiotherapists in the management of dyspareunia. Consistent with the existing literature, the results highlight ongoing challenges related to diagnostic uncertainty and focus on biomedical approaches to sexual dysfunction.

## 6. Limitations

Selection bias should be considered a limitation of this study. The survey distribution method may have favored participation by younger women due to their more frequent use of social media. Participant selection was based on subjective assumptions by the authors and included only women who provided a gynecologist’s opinion, potentially further limiting the representativeness of the sample. Relying exclusively on self-reported outcomes limits the ability to draw broader and more objective conclusions. The absence of access to medical records also prevents a comprehensive evaluation of the respondents’ medical status. Additionally, most of the recruited participants had higher education and resided in medium or large cities, which introduces an imbalance that may represent only a subset of individuals experiencing dyspareunia. Although the selection of women’s data may raise concerns about sampling bias, it should be emphasized that this mixed-methods study seeks to highlight the complexity of the diagnosis and treatment of dyspareunia, and underscores the need for further research in this field.

## Figures and Tables

**Table 1:** Questions and possible answers included in the questionnaire *.

Question	Answer
Do you consider painful intercourse to be a disease/disorder?	Yes
	No
In your opinion, is painful intercourse an embarrassing subject?	Yes
	No
How often do you have intercourse?	A few times a week
	Once a week
	Once a month
	Several times a year
	Less frequently
How often do you experience painful intercourse?	With each intercourse
	Once every 2–3 acts of intercourse
	Occasionally
Did painful intercourse begin in any of the following cases?	Since the start of intercourse
	After childbirth
	After trauma
	After surgery
Does the problem of painful intercourse negatively affect your relationship with your partner?	Yes
	No
How would you rate the pain on a scale of 1 to 10?	NRS scoring
Did you tell your gynecologist about the problem of painful intercourse?	Yes
	No
What was your gynecologist’s opinion on the issue of painful intercourse?	Short description
Have you been diagnosed with any of the following diseases/disorders?	Vaginismus
	Vulvodynia
	Endometriosis
	Chronic infections
	Hormonal disorders
	Urinary incontinence
	Pelvic organ prolapse
	I have not been diagnosed
What treatment for dyspareunia did you take?	Pharmacological
	Physiotherapeutic
	Surgery
	Psychological
	Change of sexual position
	Local lubrication

* The questionnaire was designed in Polish and then translated into English for publication.

**Table 2:** Baseline characteristics of subjects.

Variables	Subjects (*n* = 12)
Age (mean ± SD) years	26 ± 3.51
BMI (mean ± SD) kg/m^2^	21.82 ± 3.58
Pain (NRS)	7 ± 1.53
**FSFI score (mean ± SD)**	
Desire	4 ± 1.13
Arousal	4.58 ± 0.92
Lubrication	4.35 ± 1.27
Orgasm	3.73 ± 1.47
Satisfaction	5.13 ± 0.96
Pain	3.07 ± 1.3
Total score	24.86 ± 4.54
**Education**	
Higher	11 (91.7%)
Secondary	1 (8.3%)
**Residency**	
>100.000 residents	9 (75%)
>50.000 residents	2 (16.7%)
≤50.000 residents	1 (8.3%)
**Intercourse frequency**	
Once a month	3 (25%)
Once a week	5 (41.7%)
Few times a week	4 (33.3%)
**Dyspareunia frequency**	
Every intercourse	6 (50%)
Once every 2–3 intercourse	5 (41.7%)
Occasionally	1 (8.3%)
**Treatment undertook**	
Changing sexual position	8 (66.7%)
Topical lubrication	10 (83.3%)
Physiotherapy	4 (33.3%)
Pharmacotherapy	4 (33.3%)
Psychology	3 (25%)
Surgery	2 (16.7%)
Overall	11 (91.7%)

Data presented as mean standard deviation, or *n* (%).

**Table 3:** Physicians’ opinions on the reported problem of painful intercourse, classified by women according to the type of feedback.

Positive Opinion	Neutral Opinion	Negative Opinion
The problem of vulvodynia and the need for possible electroconvulsive therapy, laser therapy, and pelvic floor physiotherapy.	The presence of uterine myomas also causes a problem.	“Intercourse is possible, so from a medical point of view, there is no problem.”
Physician recommended seeing a physical therapist because there are no anatomical impediments on examination.	That the physician does not see anything disturbing.	I have only encountered disparaging opinions. That it is not a gynecological problem or that apparently it is my beauty. Of course, there was also the suggestion to change my partner for a better one (in bed, of course).
Physician concluded that treatment was necessary.	Delicate structure, shallow distribution of highly vascularized blood vessels, anteflexion of the uterus, cervical ectopy.	“You have to try. You didn’t give birth so it’s not surprising”. I was prescribed Lipodisterin for anesthesia.
Increased tone in the pelvic floor and gluteal muscles	Retroversion of uterus.	“I am going to order more tests”—after the tests, physician said he did not see anything troublesome regarding the reported issue.

## Data Availability

The raw data supporting the findings of this study can be obtained from the corresponding authors upon request.

## Supplementary Materials

The following supporting information can be downloaded at: https://www.mdpi.com/article/10.3390/jcm15020787/s1, Table S1: Characteristics of the respondents selected for the qualitative assessment.

## Abbreviations

The following abbreviations are used in this manuscript:

BMI	Body Mass Index
GSM	Genitourinary Syndrome of Menopause
FSFI	Female Sexual Function Index
NSAIDs	Nonsteroidal Anti-Inflammatory Drugs
NRS	Numerical Rating Scale
SRQR	Standards for Reporting Qualitative Research
VAS	Visual Analogue Scale
