# Supplementary material for: Exploring the Diagnostic and Therapeutic Pathways of Women with Dyspareunia: A Mixed-Methods Study
Source: J Clin Med. 2026 Jan 19;15(2):787. doi: 10.3390/jcm15020787 (PMC12841790; doi:10.3390/jcm15020787)
Supplement: Supplementary file 1 [file jcm-15-00787-s001.zip › jcm-4005522-supplementary.pdf]

Table S1. Characteristics of the respondents selected for the qualitative assessment.

Respondents/ Variables	Age (years)	BMI (kg/m²)	Education	FSFI score	Domain with the highest score	Domain with the lowest score	NRS*
**No.1**	25	18.7	Higher	24	Pain: 5.6	Lubrication: 2.8	5
**No.2**	31	17	Higher	25.3	Arousal/sexual satisfaction: 6.0	Orgasm: 1.2	7
**No.3**	22	19.03	Higher	27.8	Sexual satisfaction: 5.6	Pain: 2.8	7
**No.4**	26	24.8	Secondary	24.3	Sexual satisfaction: 5.6	Pain: 1.2	10
**No.5**	28	18.6	Higher	18.6	Sexual satisfaction: 4.8	Pain: 1.6	8
**No.6**	25	23.05	Secondary	26.8	Orgasm: 5.6	Pain: 2.8	8
**No.7**	24	22.4	Higher	31.3	Sexual satisfaction: 6.0	Pain: 4.4	7
**No.8**	24	23.2	Higher	18.6	Sexual satisfaction: 4.4	Desire: 1.8	5
**No.9**	24	21.55	Higher	28.3	Sexual satisfaction: 5.6	Pain: 3.6	6
**No.10**	35	20.2	Higher	16.6	Lubrication: 5.1	Orgasm: 3.6	9
**No.11**	24	31.1	Higher	30.5	Lubrication: 6	Orgasm: 3.6	5
**No.12**	24	22.2	Higher	26.2	Sexual satisfaction: 6	Pain: 1.6	7

* NRS used to assess pain, with a score 1-3 marked as mild, 4-6 as moderate, and 7-10 as severe pain; NRS – Numeric Rating Scale, FSFI – Female Sexual Function Index; The lower the FSFI score, the lower the quality of sexual function. The highest possible score in each domain is 6.0. The maximum total score of the questionnaire is 36.0.
